# Development of a DNA Vaccine for Melanoma Metastasis by Inhalation Based on an Analysis of Transgene Expression Characteristics of Naked pDNA and a Ternary Complex in Mouse Lung Tissues

**DOI:** 10.3390/pharmaceutics12060540

**Published:** 2020-06-11

**Authors:** Yukinobu Kodama, Mikiro Nakashima, Tadayuki Nagahara, Natsuko Oyama, Junya Hashizume, Hiroo Nakagawa, Hitomi Harasawa, Takahiro Muro, Tomoaki Kurosaki, Chikamasa Yamashita, Mitsuru Hashida, Takashi Kitahara, Hitoshi Sasaki, Shigeru Kawakami, Tadahiro Nakamura

**Affiliations:** 1 Department of Hospital Pharmacy, Nagasaki University Hospital, 1-7-1 Sakamoto, Nagasaki 852-8501, Japan; y-kodama@nagasaki-u.ac.jp (Y.K.); hassy1984-ngs@umin.net (J.H.); 07-07@umin.net (H.N.); harasawa-ngs@umin.ac.jp (H.H.); muroth1@niu.ac.jp (T.M.); kurosaki@nagasaki-u.ac.jp (T.K.); sasaki@nagasaki-u.ac.jp (H.S.); 2 Graduate School of Biomedical Sciences, Nagasaki University, 1-7-1 Sakamoto, Nagasaki 852-8588, Japan; mikirou@nagasaki-u.ac.jp (M.N.); bobby0523caldwell@gmail.com (T.N.); n.oyama.nagasaki.u@gmail.com (N.O.); skawakam@nagasaki-u.ac.jp (S.K.); 3 Faculty of Pharmaceutical Sciences, Tokyo University of Science, 2641 Yamazaki, Noda, Chiba 278-8510, Japan; chikamasa_yamashita@rs.tus.ac.jp; 4 Graduate School of Pharmaceutical Sciences, Kyoto University, 46-29 Yoshida-Shimo-Adachi-cho, Sakyo-ku, Kyoto 606-8501, Japan; hashidam@pharm.kyoto-u.ac.jp; 5 Department of Pharmacy, Yamaguchi University Hospital, 1-1-1 MinamiKogushi, Ube, Yamaguchi 755-8505, Japan; tkita@yamaguchi-u.ac.jp

**Keywords:** gene delivery, inhalation, DNA vaccine, ternary complex, melanoma

## Abstract

The present study investigated a pulmonary delivery system of plasmid DNA (pDNA) and its application to melanoma DNA vaccines. pCMV-Luc, pEGFP-C1, and pZsGreen were used as a model pDNA to evaluate transfection efficacy after inhalation in mice. Naked pDNA and a ternary complex, consisting of pDNA, dendrigraft poly-l-lysine (DGL), and γ-polyglutamic acid (γ-PGA), both showed strong gene expression in the lungs after inhalation. The transgene expression was detected in alveolar macrophage-rich sites by observation using multi-color deep imaging. On the basis of these results, we used pUb-M, which expresses melanoma-related antigens (ubiquitinated murine melanoma gp100 and tyrosinase-related protein 2 (TRP2) peptide epitopes), as DNA vaccine for melanoma. The inhalation of naked pUb-M and its ternary complex significantly inhibited the metastasis of B16-F10 cells, a melanoma cell line, in mice. The levels of the inflammatory cytokines, such as TNF-α, IFN-γ, and IL-6, which enhance Th1 responses, were higher with the pUb-M ternary complex than with naked pUb-M and pEGFP-C1 ternary complex as control. In conclusion, we clarified that the inhalation of naked pDNA as well as its ternary complex are a useful technique for cancer vaccination.

## 1. Introduction

Melanoma is one of the deadliest forms of skin cancer and its incidence is expected to increase over the next two decades. Lung metastases frequently occur and the prognosis of patients with metastatic melanoma is extremely poor [1,2,3,4]. Despite significant advances in melanoma research, effective treatments have not yet been developed for advanced melanoma [5,6].

Melanoma is among the most immunogenic cancers of all solid tumors and a number of melanoma antigens, such as glycoprotein 100, tyrosinase-related protein families, and melanoma-associated antigen families, have been reported [7,8]. Therefore, cancer vaccines have potential in the prevention and treatment of melanoma through its various surface antigens. However, traditional protein-based vaccines have been limited by the weak induction of cell-mediated immune responses [9]. Furthermore, inactivated and subunit vaccines are often impotent, stimulating suboptimal immune responses that are incapable of achieving therapeutic effects against cancer [10]. Conventional vaccine technologies have detrimental disadvantages.

DNA vaccines have recently been reported to have potential as a novel vaccine type [11]. They function by encoding protein antigens into DNA, which are delivered into cells for the production of specific proteins. DNA vaccines induce antigen-specific humoral and cell-mediated immunity [12]. They are versatile because targets may be easily modified by altering the gene sequences encoded in pDNA. They are also inexpensive, easy to manufacture on a large scale, easy to store, and safe to handle. DNA vaccines are now being investigated for various applications including cancer, infectious diseases, and autoimmune diseases [13]. However, there are currently no DNA vaccines for melanoma.

The administration route of vaccines is one of the important factors for effective and safe vaccination. Vaccines may be administered via a number of routes, including oral, intramuscular, intravenous, intraperitoneal, intradermal, and intranasal [14]. Pulmonary administration is particularly promising because the lungs contain a highly responsive immune system [15]. Pulmonary administration has been examined as a route to increase vaccine coverage due to its ease of administration and reduced risks associated with needles although the systemic administrations are safe and effective. Previous findings demonstrated the safety of this approach and showed that the pulmonary route was as efficacious as the subcutaneous route [16]. The pulmonary administration of DNA vaccines is being examined for various infections, including influenza, measles, and tuberculosis [17]. However, to the best of our knowledge, limited information is currently available on the pulmonary administration of DNA vaccines for cancer.

Although the intrapulmonary distribution of gene expression after inhalation is important information for therapeutic applications, it has not yet been elucidated in detail. Our group developed a multicolor deep imaging system using a tissue-clearing method and confocal microscopy in several organs [18,19,20,21]. We herein applied the tissue-clearing method to evaluate the intrapulmonary distribution of transgene expression using a multicolor deep imaging system for the first time.

In the present study, we compared the gene expression of naked pDNA with that of ternary complexes consisting of pDNA, dendrigraft poly-l-lysine (DGL), and γ-polyglutamic acid (γ-PGA). Ternary complexes coated with γ-PGA showed high uptake and strong gene expression in mouse dendritic cell (DC) lines [22]. We also investigated the distribution of gene expression after inhalation using the tissue-clearing method we developed and assessed the pulmonary delivery of pDNA. In addition, we evaluated the potential of a DNA vaccine using pUb-M, which expresses melanoma-related antigens (ubiquitinated murine melanoma gp100 and tyrosinase-related protein 2 (TRP2) peptide epitopes) [23,24], to prevent melanoma metastasis.

## 2. Materials and Methods 

### 2.1. Materials

pCMV-Luc, which encodes the luciferase reporter, was constructed as previously reported [25]. pUb-M was kindly provided by Prof. Reisfeld [26]. pEGFP-C1 was purchased from Clontech (Palo Alto, CA, USA). pZsGreen1-N1 was obtained from Clontech (Takara Bio Inc., Shiga, Japan).

Fifth-generation DGL compounds (MW: 172,300 Da, 963 lysine groups) were purchased from COLCOM S.A.S. (Montpellier, France). γ-PGA was kindly provided by Yakult Pharmaceutical Industry Co., Ltd. (Tokyo, Japan). Fetal bovine serum (FBS) was purchased from Biological Industries Ltd. (Kibbutz Beit Haemek, Israel). RPMI1640, antibiotics (100 U/mL penicillin and 100 µg/mL streptomycin), and other culture reagents were obtained from GIBCO BRL (Grand Island, NY, USA). All other chemicals were of the highest purity available.

Ternary complexes (the pDNA-DGL-γ-PGA complex) were constructed as previously reported [27]. The ternary complex showed a particle size of approximately 120 nm and a ζ-potential of approximately −25 mV.

### 2.2. Cells

B16-F10 cells, the mouse melanoma cell line, was obtained from the Cell Resource Center for Biomedical Research at the Institute of Development, Aging, and Cancer, Tohoku University, Japan. B16-F10 cells regularly expressing luciferase (B16-F10-Luc cells) were prepared in our laboratory as previously reported. Cells were maintained in RPMI1640 supplemented with antibiotics and 10% FBS under a humidified 5% CO_2_ atmosphere maintained at 37 °C.

### 2.3. Animals 

Five-week-old male ddY and C57BL/6N mice were purchased from Japan SLC Inc. (Shizuoka, Japan). Animal care and experimental procedures were performed in accordance with the Guidelines for Animal Experimentation of Nagasaki University with approval from the Institutional Animal Care and Use Committee (approval number: 1710191419-4 (2017)).

### 2.4. Gene Expression after the Pulmonary Administration of pDNA

Pulmonary administration was performed using the method reported previously by Yamashita et al. [28]. Ten micrograms of naked pDNA or the ternary complex containing 10 µg of pDNA were administered via the intrapulmonary route to mice at a volume of 50 µL per mouse. Mice were sacrificed 6, 24, and 48 h after administration, and the liver, kidneys, spleen, heart, and lungs were resected. These tissues were homogenized in lysis buffer (pH 7.8 and 0.1 M Tris/HCl buffer containing 0.05% Triton X-100 and 2 mM EDTA). The homogenates obtained were centrifuged at 15,000 rpm for 5 min. Ten microliters of supernatants were mixed with 50 µL luciferase assay buffer (Picagene, Toyo Ink, Tokyo, Japan) and the light produced was measured using a luminometer (Lumat LB 9507; EG and G Berthold, Bad Wildbad, Germany). Luciferase activity was indicated as relative light units (RLUs) per gram of tissue.

### 2.5. Preparation and Observation of Lung Sections

To visualize the accumulation and gene expression of the complexes, naked pEGFP-C1 and ternary complexes containing pEGFP-C1 were administered to mice by the intrapulmonary route. Twenty-four hours later, the lungs were resected. Tissue slices (thickness of 6 µm) were prepared using a microtome (REM-710; YAMATO KOHKI, Saitama, Japan). GFP expression levels in the lungs were observed using fluorescent microscopy (200× magnification, BZ-9000; KEYENCE, Osaka, Japan).

### 2.6. Tissue Clearing Method

Twenty-four hours after the pulmonary administration of naked pZsGreen1-N1 and the ternary complex containing pZsGreen1-N1, 200 µL of 0.32 mM the lipophilic carbocyanine dye, DiI (1,1′-dioctadecyl-3,3,3′,3′-tetramethylindocarbocyanine perchlorate) (42364; Sigma Aldrich, Inc., Saint Louis, MO, USA) in 5% glucose was intravenously administered, and 50 µL of 0.1 mM DiD (1,1′-dioctadecyl-3,3,3′,3′-tetramethylindodicarbocyanine perchlorate) (D307; Thermo Fisher Scientific, Waltham, MA, USA) in 5% glucose was intratracheally administered. Mice were then perfused with 4% paraformaldehyde in PBS. The lungs were immersed in Sca/e SQ reagent for 48 h [29]. Cleared lungs were observed by confocal laser scanning microscopy (200× magnification, LSM710; Carl Zeiss Microimaging GmbH, Jena, Germany).

### 2.7. Vaccination and Inhibition of Lung Metastasis 

Mice were injected with 10 µg naked pDNA, ternary complexes containing 10 µg pUb-M, or ternary complexes containing 10 µg pEGFP-C1 via the intrapulmonary route three times biweekly. Control mice were administered a 5% glucose solution via the same route. Two weeks after the last immunization, mice were administered 1 × 10^5^ B16-F10-Luc cells intravenously and lung metastasis was monitored. To evaluate lung metastasis, three weeks after the administration of B16-F10-Luc cells, tumor volumes were visualized using an in vivo imaging system (IVIS) (Caliper Life Sciences Inc.). Some mice were sacrificed, the lungs were dissected, and luciferase activities were evaluated as described above.

### 2.8. In Vivo Imaging

Three weeks after the administration of B16-F10-Luc, mice were injected intraperitoneally with 3–4.5 mg luciferin/mouse 15 min before imaging. Animals were anesthetized in a plastic chamber filled with a 2.5% isoflurane/oxygen/air mixture, and isoflurane anesthesia was maintained using a nose-cone delivery system during imaging. Pseudocolor images showing the spatial distribution of detected photon counts emerging from active luciferase within each animal were collected. Signal intensity was visualized and quantified as the sum of all detected photon counts within a region of interest using IVIS Living Image software.

### 2.9. Isolation of Splenic Cells in Mice

Spleens were dissected aseptically from mice and splenocytes were prepared using two sterilized glass slides by gently pressing spleen tissue on the rough edge of the slide to obtain lymphocytes in 10 mL of RPMI1640 medium containing 10% FBS. This cellular suspension was pipetted several times and filtered through gauze. The filtrate was then centrifuged at 1500 rpm for 5 min. Lysis buffer was added and incubated at room temperature for 15 min to lyse any trace of red blood cells. After washing, 100 µL containing 1 × 10^6^ splenocytes were prepared.

### 2.10. Evaluation of Antigen-Specific Cytokine Secretion

To prepare tumor cell lysates (B16-F10 cells), cells were scraped from the plates and suspended in culture medium. Two weeks after the last immunization, splenic cells collected from immunized mice were plated on 96-well plates and incubated at 37 °C for 72 h in the presence or absence of 100 µL of tumor cell lysates containing 1 × 10^5^ cells. IFN-γ, TNF-α, and IL-6 in the culture medium were measured using a suitable commercial ELISA kit (eBioscience Co., Ltd., San Diego, CA, USA).

### 2.11. Statistical Analysis

Multiple comparisons were performed using Tukey’s or Dunnett’s pairwise multiple comparison t-test. *p* < 0.05 was considered statistically significant.

## 3. Results

### 3.1. Gene Expression after the Pulmonary Administration of Naked pDNA and the Ternary Complex

**Figure 1:**
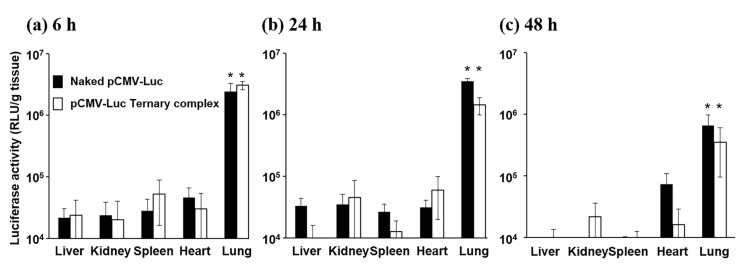
In vivo transfection experiments. Mice were administered naked pDNA or the pCMV-Luc ternary complex, sacrificed 6 (**a**), 24 (**b**), and 48 h (**c**) later, and the liver, kidneys, spleen, heart, and lungs were then dissected. Gene expression was assessed as luciferase activity. Data are the mean ± S.E. (*n* = 3). * *p* < 0.05 (Tukey’s test).

Mice were administered naked pCMV-Luc and ternary complexes containing pCMV-Luc by the intrapulmonary route and their transgene efficiencies in organs, such as the liver, kidneys, spleen, heart, and lungs, were evaluated 6, 24, and 48 h after administration (Figure 1). Luciferase activity in the lungs 6 and 24 h after administration of naked pDNA and the ternary complex was significantly higher than that in other organs.

**Figure 2:**
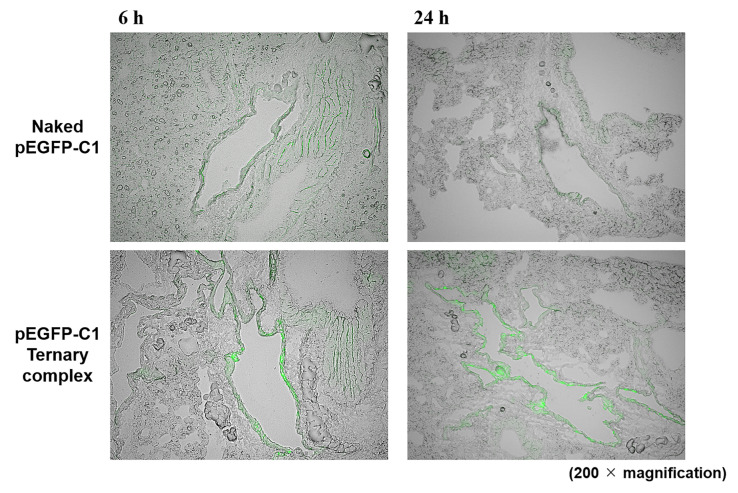
Preparation and observation of lung sections. Naked pDNA and the pEGFP-C1 ternary complex were administered by the intrapulmonary route to mice. Mice were sacrificed 6 or 24 h later and the lungs were harvested and processed. Fluorescence was visualized using fluorescent microscopy.

To assess intrapulmonary gene expression in more detail, naked pEGFP-C1 and the ternary complex containing pEGFP-C1 were administered to mice by the intrapulmonary route and the lungs were dissected 6 or 24 h later. Strong fluorescence tended to be localized within the respiratory epithelium 6 and 24 h after the administration of the ternary complex, while weak fluorescence was observed in whole lungs from mice administered naked pEGFP-C1 (Figure 2). Furthermore, lungs from mice administered naked pEGFP-C1 and the ternary complex containing pEGFP-C1 did not show macroscopic damage (data not shown).

### 3.2. Spatial Distribution of Naked pDNA and the Ternary Complex

**Figure 3:**
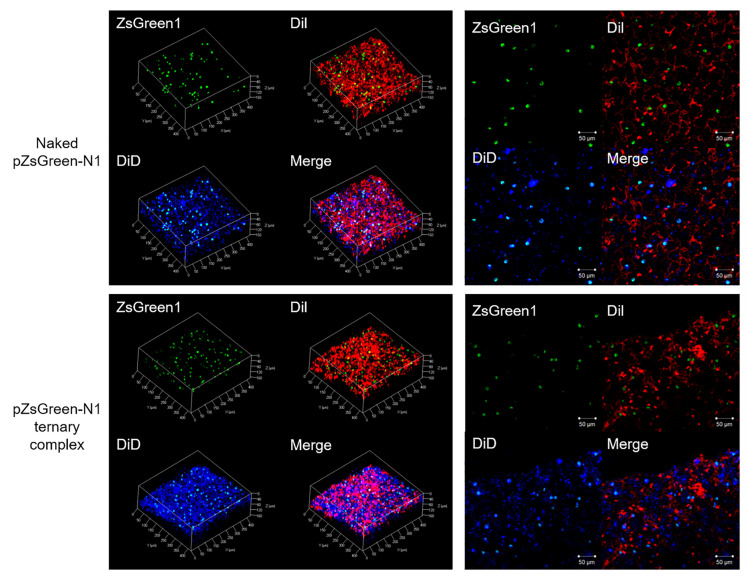
Spatial distribution of gene expression. Naked pZsGreen1-N1 and the pZsGreen1-N1 ternary complex were administered to ddY mice via the intrapulmonary route. Mice were sacrificed 24 h later and the lungs were subjected to the tissue cleaning method. Fluorescence was observed by confocal laser scanning microscopy. Green: ZsGreen1 expression, Red: DiI-stained vascular cells, and Blue: DiD-stained surface cells of the alveoli.

To clarify the dispersibility of transgene expression, the lungs were prepared for the multicolor deep imaging system using the tissue clearing reagent Scale SQ. We found that intravenously administered DiI stained vascular cells and consequently the structure of alveolar could be visualized by staining capillaries. On the other hand, we found that intratracheally administered DiD stained the stained alveolar membrane, and consequently thin and flat alveolar epithelial type I cells were lightly stained, and alveolar macrophage or alveolar epithelial type II cells were strongly stained. From these results, we thought that the distribution of foreign gene expression in lung tissue could be achieved using this novel staining method. Green fluorescence and blue fluorescence were observed to overlap. Thus, ZsGreen1 expression was mainly observed in alveolar macrophage and/or alveolar epithelial type II cells-rich sites in the lungs for both naked pDNA and the ternary complex (Figure 3).

### 3.3. Suppressive Effects on Melanoma Metastasis after the Pulmonary Administration of Naked pDNA and the Ternary Complex

**Figure 4:**
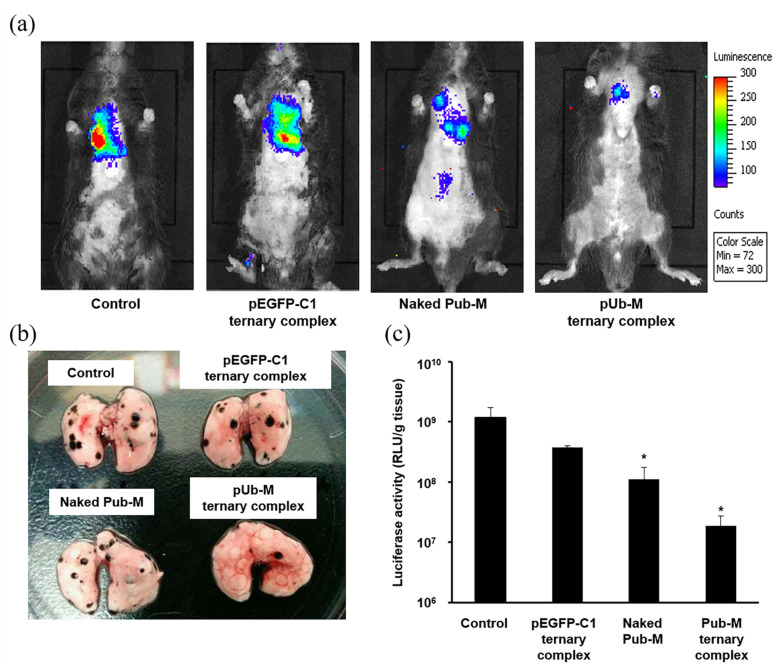
Cancer vaccine effects against lung metastatic tumors by DNA vaccination. Suppressive effects on pulmonary metastatic tumors were evaluated by DNA vaccination using control, naked pUb-M, the pUb-M ternary complex, or pEGFP-C1 ternary complex (50 µg of pDNA). Two weeks after the last immunization, B16-F10-Luc cells (for the evaluation of tumor metastasis) were injected intravenously (1 × 10^5^ cells) into mice. Pulmonary metastatic tumors three weeks after the tumor injection were evaluated by IVIS imaging (**a**) and luciferase activity (**c**) (*n* = 3, each value represents the mean ± S.E.). * *p* < 0.05, vs. control. Photograph of a B16-F10-derived pulmonary metastatic tumor three weeks after the tumor injection in mice immunized by each transfection method (**b**).

The ternary complex containing pUb-M (Pub-M complex) was administered to mice by the intrapulmonary route and immune responses against B16-F10 cells were evaluated as the melanoma metastatic model. Tumor growth following lung metastasis was visualized by measuring fluorescent intensity with IVIS imaging (Figure 4a). Tumor growth was suppressed in the Pub-M complex and naked pUb-M, as indicated by both tumor volume and luciferase activity. No significant differences were observed among the control group and the ternary complex containing pEGFP-C1 (the pEGFP-C1 complex). In the metastatic model, naked pUb-M and the Pub-M complex inhibited the lung metastasis of B16-F10 cells visually and luciferase activity was reduced (*p* < 0.05, Figure 4b,c).

### 3.4. Antigen-Stimulatory Cytokine Secretion from Splenic Cells after the Pulmonary Administration of Naked pDNA and the Ternary Complex

**Figure 5:**
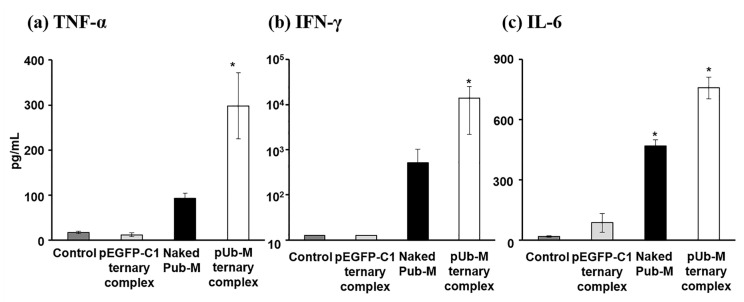
Cytokines secreted from splenic cells of immunized mice. Melanoma-stimulatory cytokine secretion characteristics by DNA vaccination using the complex constructed with pUb-M. Cancer cell lysate-specific IFN-γ, TNF-α, and IL-6 secretion from splenic cells immunized three times biweekly with control, naked pUb-M, the pUb-M ternary complex (50 µg of pDNA), or pEGFP-C1 ternary complex (50 µg of pDNA). Splenic cells (5 × 10^5^ cells) were collected two weeks after the last immunization. After immunized splenic cells were cultured for 72 h in the presence of a cancer cell lysate (5 × 10^4^ cells), IFN-γ, TNF-α, and IL-6 secreted in the medium were measured by ELISA. Each value represents the mean ± S.E. (*n* = 3). * *p* < 0.05, vs. control.

To evaluate melanoma-specific cytokine secretion from immunized splenic cells, splenic cells immunized by pUb-M were incubated with a tumor cell lysate in vitro, and cytokines secreted into the supernatants were measured. Splenic cells immunized by the Pub-M complex secreted the largest amounts of IFN-γ, TNF-α, and IL-6 in the presence of B16-F10 cell lysates (Figure 5).

## 4. Discussion

The lungs contain abundant amounts of immunocompetent cells, such as alveolar macrophages, which are the first line of defense against pollutants and pathogenic microbes that initiate an innate immune response in the lungs. The lungs are also a deliverable tissue directly via devices, have a large surface area, and are highly cellular and vascularized to facilitate transfection [30,31]. The lungs are a potential site for the delivery of DNA vaccines. Pulmonary administration is a desirable route due to its non-invasive nature, increased local drug concentration, and minimal systemic side effects. Although pulmonary mucosal vaccination represents a very promising approach, research in this area is still in its infancy. In the present study, we examined the utility of pulmonary administration for the delivery of pDNA to the lungs.

We used pCMV-Luc to evaluate transfection efficacy and examined gene expression in each organ after the pulmonary administration of naked pDNA and the ternary complex. Naked pDNA showed strong gene expression in the lungs (Figure 1). This result is in contrast to the generally accepted theory that naked pDNA achieves weak gene expression because of poor intracellular uptake and degradation by endonucleases. However, Ito et al. previously reported that the inhalation of naked pDNA powder composed of hyaluronic acid achieved strong gene expression in the lungs [32]. Furthermore, an RNA interference (RNAi) effect was demonstrated following the inhalation of naked siRNA solution [33]. The lung has many pharmacokinetic-anatomical advantages as a drug administration site [34,35], and these pharmacokinetic-anatomical characteristics may contribute to the strong transfection of naked pDNA after inhalation.

An important factor for effective gene therapy is the delivery of the therapeutic gene to the target site. However, transgene expression in lung tissues after the inhalation of naked pDNA and its complex has not yet been elucidated in detail. Therefore, we attempted to examine the distribution of transgene expression after the pulmonary administration of naked pEGFP-C1. GFP was observed in entire lung sections (Figure 2). On the other hand, the data of any lung section can observe only a part of the lung. Then, we clarified the tissue distribution at the tissue level by newly introducing the tissue clearing method shown in Figure 3. The results of the spatial distribution study revealed that transgene expression was abundant in the alveolar macrophage- and alveolar epithelial cell-rich sites of the lungs after the pulmonary administration of naked pDNA (Figure 3). These results suggest that the pulmonary administration of naked pDNA is suitable for vaccines.

The induction of adaptive immunity by DNA vaccines needs to efficiently transfect antigens to antigen-presenting cells (APCs), such as macrophages and DCs. In recent years, pDNA carriers for DNA vaccination have emerged as one of the most promising strategies for antigen delivery to APCs. Furthermore, pDNA carriers protect DNA vaccines from degradation and significantly improve both humoral and cellular immune responses. To date, a number of vaccine vectors have been developed, including polymer nanoparticles and liposomes. We developed polymer nanoparticles as vaccine vectors and reported that γ-PGA-coated nanoparticles showed abundant accumulation and strong gene expression in the marginal zone of the spleen after intravenous administration [24]. Furthermore, γ-PGA-coated nanoparticles containing pUb-M inhibited tumor growth and suppressed lung metastasis after intravenous administration [24]. Based on our previous findings, we herein hypothesized that the inhalation of the ternary complex (pDNA-DGL-γ-PGA complex) may be efficiently transfected in lung tissues and this formulation may be a new pDNA delivery platform for DNA vaccination to prevent melanoma metastasis. Although gene expression sites of the ternary complex in the lungs differed to that of naked pDNA (Figure 2), both naked pDNA and the ternary complex showed high gene expression in alveolar macrophage and/or alveolar epithelial type II cells-rich sites in lungs (Figure 3). Anionic particles generally are not taken up by cells because they repulse the cellular membrane electrostatically. Several anionic polymers, however, have been reported taken up by endothelial cells and macrophages via specific receptors such as scavenger receptor and toll-like receptor (TLR) [36,37]. As well, γ-PGA induces immunity through specific receptors, such as TLR4, which is expressed in DCs and macrophages [38,39]. In addition, Uto et al. reported that γ-PGA nanoparticles induced pro-inflammatory cytokine production via TLR4 signaling pathways in mouse macrophages [40]. The ternary complex showed a stronger vaccine effect than naked pDNA, suggesting that the ternary complex can be efficiently taken up by alveolar macrophages than naked pDNA. These results strongly suggest that pulmonary administration is a suitable route for the application of DNA vaccines not only for naked pDNA, but also the ternary complex.

To assess immune induction effects after the pulmonary administration of the DNA vaccine, we examined the suppression of lung metastasis by melanoma using pUb-M as a model antigen. pEGFP-C1 complex appears to slightly inhibit lung metastasis of B16-F10, although not significantly (Figure 4c). The pDNA was reported to induce inflammatory reaction through toll-like receptor [41]. These inflammatory reactions might cause the weak tumor inhibitory reaction. Naked pUb-M inhibited the lung metastasis of B16-F10 cells (Figure 4). The naked pDNA formulation does not cause systemic toxicity or cytotoxicity due to vaccine carriers. Cytotoxicity derived from some carriers on the lung epithelial surface has prevented the clinical application of gene inhalation therapy [42]. A previous study also reported that nanoparticles did not appear to be suitable for direct administration to the lungs because of cytotoxicity caused by cationic charges [43]. Toxicity in the lungs may cause lethal diseases, such as acute lung injury (ALI) and acute respiratory distress syndrome (ARDS); therefore, cationic nanoparticles cannot be appropriated for inhalation [44,45,46]. We consider the inhalation of naked pDNA to be a simple and safe method for vaccination. On the other hand, the cytotoxicity to lungs caused by particles is due to their cationic charge, while the ternary complex in this study has anionic surface charge and constructed with only biodegradable compounds. In fact, there were no mice which died or were weakened after inhalation of ternary complex. This result is consistent with our previous findings on intravenous administration showing that the ternary complex was not cytotoxic due to its anionic charge [27]. In addition, the ternary complex (pUb-M complex) more strongly inhibited the lung metastasis of B16-F10 cells than naked pUb-M (Figure 4). These results are consistent with transfection characteristics in lung tissues (Figure 2). Therefore, the vaccine effects of the ternary complex may be stronger than those of naked pUb-M. The inhalation of not only naked pDNA, but also the ternary complex may be a safe method under these experimental conditions for DNA vaccination. We will examine the detailed lung toxicity of intratracheally administered ternary complex in future experiments based on the results of this study.

To achieve potent therapeutic effects by DNA vaccination against cancer, the activation of Th1 immunity is important [47]. In the present study, splenic cells immunized by naked pUb-M secreted abundant amounts of IFN-γ, a Th1 cytokine. These results suggest that inhaled naked pDNA exhibits immunostimulatory activity. On the other hand, the secretion of Th1 cytokines (IFN-γ and TNF-α) by splenic cells immunized by the pUb-M complex was significantly greater than those immunized by naked pUb-M (Figure 5). Since these differences between naked pUb-M and the pUb-M complex are consistent with transfection characteristics in lung tissues, the pUb-M complex appeared to have been effectively taken into the APCs of AECs. Effective DNA vaccine effects for the prevention of melanoma metastasis by not only naked pDNA, but also the ternary complex may have involved the enhanced secretion of Th1 cytokines as one of the underlying mechanisms. Further study such as the protein expression level of these cytokines in sera and tumor tissues, the infiltration of immune cells will be necessary in the future.

## 5. Conclusions

We herein elucidated transgene expression characteristics in lung tissues by inhaled naked pDNA (pCMV-Luc, pEGFP-C1, and pZS-Green-N1) and these ternary complexes. We also demonstrated that inhaled naked pUb-M and the pUb-M ternary complex efficiently prevented melanoma metastasis as a novel technology for DNA vaccination. The inhalation of not only naked pUb-M, but also the pUb-M ternary complex represents a safe method under these experimental conditions for DNA vaccination. Therefore, the delivery system by naked pUb-M as well as the pUb-M ternary complex by inhalation has potential as a vaccine platform to protect against melanoma metastasis. Although further studies are needed to clarify the underlying mechanisms, the present results will provide valuable information on the development of DNA vaccine formulations for melanoma metastasis.
